# Vulnerable and Grandiose Narcissism Are Differentially Associated With Ability and Trait Emotional Intelligence

**DOI:** 10.3389/fpsyg.2018.01606

**Published:** 2018-08-28

**Authors:** Marcin Zajenkowski, Oliwia Maciantowicz, Kinga Szymaniak, Paweł Urban

**Affiliations:** Faculty of Psychology, University of Warsaw, Warsaw, Poland

**Keywords:** grandiose narcissism, vulnerable narcissism, emotional intelligence, ability, narcissism

## Abstract

We examined the association between two types of narcissism, grandiose and vulnerable, and self-reported as well as ability emotional intelligence (EI). Grandiose narcissism is characterized by high self–esteem, interpersonal dominance and a tendency to overestimate one’s capabilities, whereas vulnerable narcissism presents defensive, avoidant and hypersensitive attitude in interpersonal relations. In the current study (*n* = 249) we found that vulnerable narcissism was significantly and negatively associated with trait (self-reported) EI; however, it did not correlate with ability (performance) EI. Grandiose narcissism was significantly positively connected with trait EI. Moreover, when the two EI scores were analyzed together in a single model, they were associated with grandiose narcissism in opposite directions. Specifically, trait EI showed a positive relation with grandiose narcissism, while ability EI negatively predicted this type of narcissism. The latter results are consistent with previous findings showing that individuals with high level of grandiose narcissism tend to overestimate their abilities. Vulnerable narcissism is probably connected with more realistic self-perception of emotional abilities.

## Introduction

Nowadays, an increasing tendency to describe narcissism as a non–clinical personality trait is being observed among psychologists (e.g., Paulhus and Williams, 2002). Empirical data show that narcissism is connected to a variety of psychological variables such as aggression (e.g., Krizan and Johar, 2015), self–esteem and well–being (e.g., Sedikides et al., 2004; Dufner et al., 2012). Several studies explored also the relationship between narcissism and constructs related to emotional functioning, such as empathy and emotional intelligence (EI). However, these studies provide rather mixed results. Whereas some researchers found narcissism to be associated with low empathy (Delič et al., 2011), others reported no relation, or a positive correlation between narcissism and empathy (e.g., Jonason and Kroll, 2015). Likewise, in some cases narcissism was positively associated with EI (Petrides et al., 2011; Veselka et al., 2012; Nagler et al., 2014; Zhang et al., 2015), while in other studies this relationship was close to zero or even negative (Vonk et al., 2013; Austin et al., 2014; Czarna et al., 2016; Jauk et al., 2016). The aim of the present study was a deeper understanding of the association between narcissism and EI. A careful analysis of prior work presented below reveals that the ambiguous findings might be related to the fact that both narcissism and EI are complex constructs and their relationship depends on the specific aspect being analyzed (e.g., type of narcissism) or the conceptualization and assessment method (e.g., self-report vs. performance EI).

### Grandiose and Vulnerable Narcissism

Some researchers suggest that narcissism might not be a unitary construct. The distinction between vulnerable and grandiose narcissism was made by Wink (1991). The two forms of narcissism share several characteristics such self-centeredness, exaggerated sense of self-importance and entitlement, disagreeableness, and a tendency to interact with others in an antagonistic manner (Dickinson and Pincus, 2003; Miller et al., 2011). Regardless of the narcissistic common core, each dimension has its own exclusive characteristic. Individuals with high vulnerable narcissism are described as being defensive, avoidant, insecure, hypersensitive and vigilant for criticism (Wink, 1991; Miller et al., 2011). At the same time they need people’s recognition (e.g., admiration) to bolster their self–worth. Feeling underestimated may result in withdrawal and passive attitude in interpersonal relations (Pincus et al., 2009; Miller et al., 2011). Vulnerable narcissism is also associated with lower levels of self esteem, extraversion and agreeableness, higher neuroticism (Miller et al., 2011, 2018; Maciantowicz and Zajenkowski, 2018), a negative view of the past and fatalistic attitude (Zajenkowski et al., 2016).

Grandiose narcissism is characterized by high self–esteem, interpersonal dominance and tendency to overestimate one’s capabilities (Wink, 1991; Pincus et al., 2009; Miller et al., 2011). Individuals with high grandiose narcissism tend to endorse positive illusions about themselves, simultaneously repressing information inconsistent with an inflated self-image (Campbell and Foster, 2007). They fantasize about superiority, perfection, omnipotence. Grandiosity can also be manifested through exploitativeness and aggressive behaviors (Pincus et al., 2009). Grandiose narcissism negatively correlates with neuroticism and agreeableness, and positively with extraversion (Miller et al., 2011). Several studies revealed a tendency to overestimate one’s own cognitive ability among people scoring high on grandiose narcissism (Gabriel et al., 1994; Paulhus and Williams, 2002; Zajenkowski and Czarna, 2015).

### Ability and Trait Emotional Intelligence and Their Association With Narcissism

Emotional intelligence was defined by Salovey and Mayer (1990, p.189) as the *ability to monitor one’s own and others’ feeling and emotions, to discriminate among them and to use this information to guide one’s thinking and actions*. In their model four branches have been distinguished: *Perception of Emotions* (the ability to identify one’s emotions accurately, as well as to recognize emotions of other people based on various contextual cues), *Using Emotions to Facilitate Thinking* (the ability to use emotions and moods to support and guide intellectual processing), *Understanding emotions* (skills necessary to comprehend and label basic and complex emotions), *Managing Emotions* (the ability to monitor and modify own emotions in order to enhance emotional and intellectual growth). Within this approach EI is measured similarly to cognitive intelligence via performance tests (Mayer et al., 2003). In another popular model, EI is defined as people’s perceptions of their emotional world, or a constellation of emotional self-perceptions located at the lower levels of personality hierarchies (e.g., Petrides et al., 2007; Petrides et al., 2011). It is believed that one’s perception of emotional effectiveness is, at least partially, associated with genuine emotional skills (e.g., Van der Linden et al., 2017). In this approach, EI is assessed via rating scales and self-report questionnaires. It need to be acknowledged that in the research literature EI based on performance tests is typically labeled ‘ability EI’, whereas self-reported EI is often labeled ‘trait EI’ (e.g., Zeidner et al., 2009). In the current article we use this terminology.

To date, a few studies have examined the relationship between EI and narcissism, with the latter being mainly considered in the grandiose version (Petrides et al., 2011; Vonk et al., 2013; Austin et al., 2014; Nagler et al., 2014; Zhang et al., 2015; Czarna et al., 2016; Jauk et al., 2016). The empirical data in this area are rather ambiguous; however, a deeper analysis of existing findings provides some general observations. In **Table 1** we present previous studies linking narcissism with EI. First, in most studies using self-report EI measures (e.g., Trait EI Questionnaire by Petrides and Furnham, 2006; The EI Scale by Schutte et al., 1998) a positive correlation with grandiose narcissism has been reported (Petrides et al., 2011; Vonk et al., 2013; Austin et al., 2014; Nagler et al., 2014; Zhang et al., 2015); though there were studies with no significant association (study 2 by Munro et al., 2005; Austin et al., 2014; Jauk et al., 2016). The ability measures of EI (e.g., Mayer Salovey Caruso EI Test by Mayer et al., 2003; The Test of EI by Śmieja et al., 2014) exhibit much weaker correlations with grandiose narcissism, hardly reaching significance level (−0.16 in Zhang et al., 2015; -0.06 in Czarna et al., 2016; -0.11 in Jauk et al., 2016). It is also worth mentioning that one recent study explored the tendency of individuals with high grandiose narcissism to overestimate their EI (Lobbestael et al., 2016). It has been found that, similarly to cognitive abilities, those scoring high on grandiose narcissism show inflated views of their emotional abilities. However, this study used a measure assessing mentalizing abilities rather than global EI.

**Table 1:** Articles reporting correlation between narcissism and emotional intelligence.

Article	Narcissism measure	EI measure	*N*	Sample	Correlation coefficients	Country
**Vulnerable Narcissism**		
Austin et al. (2014)	HSNS	TEIQue	369, 432	students	−0.48^∗∗∗^; −0.44^∗∗^	United Kingdom
Vonk et al. (2013)	PNI	EIS	368	Students	−0.19^∗∗∗^	United States
**Grandiose Narcissism**		
Austin et al. (2014)	NPI−16	TEIQue	369, 432	students	0.21^∗∗∗^; 0.06	United Kingdom
Czarna et al. (2016)	NPI	TIE (performance)	273	students	0.06	Poland
Delič et al. (2011)	NPI	ESCQ	306	students	0.38^∗∗^	Slovenia
Jauk et al. (2016)	DTDD	TEIQue	540	students	0.09	Germany
Jauk et al. (2016)	DTDD	MSCEIT (performance)	540	students	−0.26^∗∗^	Germany
Munro et al. (2005)	NACE	EIS	237	students	−0.11	Australia
Nagler et al. (2014)	NPI 17	SSI	594	students	Emotional expressivity: 0.15^∗∗∗^ Emotional sensitivity: −0.04 Emotional control: 0.15^∗∗∗^ Emotional manipulation: 0.69^∗∗∗^	Austria, Germany
Petrides et al. (2011)	NPI	TEIQue	214	adult twin pairs	TEIQue: 0.23^∗^ Emotion expression: 0.15 Emotion management: 0.38^∗^ Emotion perception: 0.18^∗^ Emotion regulation: 0.04	United Kingdom
Vonk et al. (2013)	NPI, PNI	EIS	368	students	PNI: 0.14^∗∗^ NPI Exhibitionism: −0.01 NPI Entitlement/Exploitativeness −0.14^∗∗^ NPI Leadership/Authority: 0.14^∗∗^	United States
Zhang et al. (2015)	NPI	SEIS	396	adolescents	0.34^∗∗∗^	China
Zhang et al. (2015)	NPI	MSCEIT (performance)	396	adolescents	−0.16^∗∗^	China

*^∗^p < 0.05. ^∗∗^p < 0.01. ^∗∗∗^p < 0.001. MSCEIT, Mayer–Salovey– Caruso Emotional Intelligence Test; TIE, Test Inteligencji Emocjonalnej (The Test of Emotional Intelligence). TEIQue, Trait Emotional Intelligence Questionnaire; EIS, Emotional Intelligence Scale; ESCQ, Emotional Skills and Competence Questionnaire; SSI, Social Skills Inventory; NPI, Narcissistic Personality Inventory; PNI, Pathological Narcissism Inventory; NACE, Narcissism – Aloofness –Confidence – Empathy scale; DTDD, Dark Triad Dirty Dozen; HSNS, Hypersensitive Narcissism Scale.*

In the case of vulnerable narcissism, there is less empirical evidence regarding its relation with EI. Actually, we found only two studies, both reporting a negative correlation between vulnerable narcissism and self-report EI (Vonk et al., 2013; Austin et al., 2014).

### The Current Study

In the current study we examined, for the first time, the association between both types of narcissism and different conceptualizations of EI. Specifically, our research encompassed measures of vulnerable and grandiose narcissism as well as trait (self-report) and ability (performance) EI. Below, we formulate predictions based on the previous studies and theoretical considerations.

When it comes to vulnerable narcissism, the existing empirical data (Vonk et al., 2013; Austin et al., 2014) suggest that it should be negatively correlated with trait EI (H1). Moreover, vulnerable narcissists have a tendency toward negative self-views (e.g., Miller et al., 2011). In the case of ability EI, we expect similar tendency (an inverse relationship; H2); however, this expectation is speculative in nature and based mostly on the common correlates of both constructs. Studies show that ability EI is positively related to empathy (Ciarrochi et al., 2000) while the latter is associated with low level of vulnerable narcissism (Lannin et al., 2014). Moreover, Lopes et al. (2005) found that emotion regulation abilities are connected to prosocial tendencies, which are rather foreign to people with a high level of vulnerable narcissism (e.g., Miller et al., 2011). Additionally, such characteristics of vulnerable narcissism as emotional instability, concentration on self (eg., Wink, 1991; Miller et al., 2011), difficulties in sustaining relationships, and taking others’ perspective (eg., Vonk et al., 2013) are typically related with low ability EI (Mayer et al., 2004).

In the case of grandiose narcissism, its relation with EI seems to be much more puzzling. As noted above, in most studies this type of narcissism was positively correlated with self-report EI (Petrides et al., 2011; Vonk et al., 2013; Austin et al., 2014; Nagler et al., 2014; Zhang et al., 2015). Moreover, people with a high level of grandiose narcissism have been found to overestimate their abilities, including emotional skills (Lobbestael et al., 2016). Indeed, socio-emotional skills are regarded as desirable in society (e.g., Czarna et al., 2016) and may be associated with an inflated narcissistic self-image (e.g., Miller et al., 2011). Thus, we hypothesized that grandiose narcissism will be positively associated with trait EI (H3).

The relationship between grandiose narcissism and ability EI seems the most difficult to predict. Some research results indicate that grandiose narcissism might be related to better performance on tasks assessing theory of mind, and higher self-report empathy (Delič et al., 2011; Vonk et al., 2013; Jonason and Kroll, 2015). Moreover, individuals with a high level of grandiose narcissism tend to manipulate and exploit other people, which suggests that their emotional competencies might be high (Nagler et al., 2014). Thus, these findings would suggest a positive association of grandiose narcissism with ability EI. However, the existing studies directly linking these two constructs show slightly negative, but rather marginal correlation (Zhang et al., 2015; Czarna et al., 2016; Jauk et al., 2016). Taking all these considerations together, we believe that there is no sufficient evidence to postulate a clear hypothesis here.

Finally, a question arises about the congruence between narcissists’ self-views and their actual abilities. Taking into account the inflated self-views and a tendency to overestimate abilities in grandiose narcissism, we expected that grandiose narcissism will be positively associated with trait EI controlling for participants’ ability EI (H4). In the case of vulnerable narcissism, we expected that it will be negatively related to trait EI and that this association will be attenuated controlling for ability EI (H5). It needs to be acknowledged, however, that H4 and H5 are more exploratory, because the existing research literature does not provide direct evidence for these expectations.

## Materials and Methods

All data were uploaded to Open Science Framework and are available under the following address: osf.io/j9yqr.

### Participants

The study involved 249 volunteer participants (174 female, 75 male) recruited via publicly accessible social networking websites. The mean age of the sample was 22.67 years (*SD* = 5.42). It was composed of undergraduate students (61.4%) and individuals holding a Master’s degree (38.6%). Study was conducted using an online survey. This sample size allowed to detect effects of correlation of 0.20 with the power equal 0.90. This study, including the consent process meets the standards of the ethics committee of Faculty of Psychology at University of Warsaw. Informed consent was obtained from all participants. Participation was voluntary and participants were allowed to reject or withdraw at any point with no disadvantage to their treatments.

### Measures

#### Hypersensitive Narcissism Scale

To assess vulnerable narcissism we used the Hypersensitive Narcissism Scale (HSNS) by Hendin and Cheek (1997) in the Polish version (Czarna et al., 2014). This measure is composed of 10 items with a five point Likert-like response scale from 1 (*strongly disagree*) to 5 (*strongly agree)*, e.g., *My feelings are easily hurt by ridicule or the slighting remarks of others; When I enter a room I often become self-conscious and feel that the eyes of others are upon me.* The sum of items creates an index of vulnerable narcissism. In the present sample α = 0.73.

#### Narcissistic Personality Inventory

Grandiose narcissism was assessed with the Narcissistic Personality Inventory (NPI; Raskin and Hall, 1979). We used the validated Polish adaptation of the NPI (Bazińska and Drat-Ruszczak, 2000), which contains 34 items. The items were selected basing on the factor analysis (loadings exceeding 0.40) and represented the following subscales of the NPI: Authority (11 items), Self-Sufficiency (7), Vanity (5), and Exhibitionism (11). Respondents rated their degree of endorsement of each statement using a 5-point Likert-like response format, from 1 (*does not apply to me*) to 5 (*applies to me*), e.g., *I like to be the center of attention; I like to be complimented*. The sum of items creates an index of grandiose narcissism. NPI achieved high levels internal consistency (α = 0.93).

#### The Test of Emotional Intelligence

Ability EI was measured with The Test of EI (*Test Inteligencji Emocjonalnej*, TIE; Śmieja et al., 2014). The measure is based on Salovey and Mayer (1990) concept of EI and consists of four subscales: Perception, Understanding, Facilitation, and Management of emotions. Each of them is comprised of six item parcels. Each parcel describes one situation posing an emotional problem, in which there are three possible alternatives.

TIE is divided into two parts. In the first one (Perception and Understanding) respondents are asked to evaluate the probability of experiencing alternative emotions by the person involved in a situation on a 5-point Likert scale, from 1 (*very bad answer*) to 5 (*very good answer*). In the second part (Facilitation and Management*)* participants again use a 5-point Likert-like response format, from 1 (*very ineffective*) to 5 (*very effective*), to judge the level of appropriateness of three possible actions that may be taken to solve the problem, e.g.:

*Sophie hits the table with a fist. She frowns, her face is glowing, and her teeth are clenched. Most probably:*

- *She is watching a popular show on TV*
  1......2......3......4......5
- *Once again she hurt her finger while cutting bread*
  1......2......3......4......5
- *She was just told by a colleague that he will not help her to prepare an important project, because he is leaving for a last-minute holiday*
  1......2......3......4......5

The similarity of a testee’s responses with the answers given by the professionals is the basis of the final scoring. The group of experts was comprised of psychotherapists, HR specialists and trainers. There are two final results (sum of items), one for each of the branches and one for the whole test. TIE shows high overall reliability (α = 0.88).

#### Trait Emotional Intelligence Questionnaire – Short Form

We used The Trait EI Questionnaire – Short Form (TEIQue – SF; Petrides and Furnham, 2006) adapted into Polish by Wytykowska and Petrides (2007) to measure self–assessed EI. The scale, based on the full form of the TEIQue, has 30 items ranked on a scale ranging from 1 (*completely disagree)* to 7 (*completely agree*), e.g., *Many times, I can’t figure out what emotion I’m feeling; Expressing my emotions with words is not a problem for me*. The sum of items creates a total score of trait EI. The internal consistency was high (α = 0.79).

Measures were administered in the following order: NPI, HSNS, TEIQue – SF, TIE.

### Analyses

In order to test H1 – H3 Pearson’s correlations between narcissisms and EI measures were calculated. Furthermore, series of regression analyses were conducted to examine how each of the EI measure is uniquely associated with grandiose and with vulnerable narcissism (tests of H4 and H5).

## Results

In **Table 2** we present correlations and descriptive statistics of all variables. Both grandiose and vulnerable narcissism highly correlated with trait EI; however, in opposite directions: positive (0.49; *p* < 0.001) and negative (−0.55, *p* < 0.001), respectively. Thus H1 and H3 were confirmed. Neither grandiose (−0.11, *p* = 0.077) nor vulnerable narcissism (−0.12, *p* = 0.068) were significantly related to the general score of the ability EI; however they did correlate with the TIE subscales. Specifically, vulnerable narcissism negatively correlated with Facilitation (−0.24, *p* < 0.001) and Management (−0.13, *p* = 0.046), which partially confirms H2. Grandiose narcissism was negatively connected to the Understanding subscale (−0.15, *p* = 0.017).

**Table 2:** Correlations and descriptive statistics for all variables (*N* = 249).

	1	2	3	4	5	6	7	8
1 Vulnerable narcissism								
2 Grandiose narcissism	0.03							
3 Ability EI Total	−0.12	−0.11						
4 Ability-perception	−0.07	−0.08	0.80***					
5 Ability-understanding	0.10	−0.15*	0.70***	0.49***				
6 Ability-facilitation	−0.24***	0.00	0.74***	0.46***	0.25***			
7 Ability-management	−0.13*	−0.11	0.73***	0.39***	0.35***	0.47***	–	
8 Trait EI	−0.55***	0.49***	0.15*	0.12	−0.03	0.23***	0.12	–
*M*	31.92	101.16	29.50	8.06	7.82	7.00	6.62	4.35
*SD*	6.33	22.94	3.93	1.42	1.28	1.33	1.24	0.88
α	0.73	0.93	0.79					0.79

*^∗^p< 0.05, ^∗∗^p< 0.01, ^∗∗∗^p< 0.001.*

Subsequently, we conducted a series of regression analyses in order to test H4 and H5 and for deeper understanding of the obtained results. First, we examined the unique contribution of each EI measure for both types of narcissism. We conducted two regressions with both EIs analyzed jointly as predictors and each narcissism as a dependent variable (see **Table 3**). The analyses revealed that trait EI explained more variance than ability EI in both types of narcissism. Moreover, ability EI taken together with trait EI became significant in predicting grandiose narcissism. Interestingly, the two EI measures showed opposite directions of associations: trait was positively (β = 0.52, *p* < 0.001) and ability was negatively (β = -0.19, *p* = 0.001) related to grandiose narcissism. In the case of vulnerable narcissism, ability EI (β = -0.04, *p* = 0.496) remained a non-significant predictor when analyzed together with trait EI (β = -0.54, *p* < 0.001).

**Table 3:** Regression analyses with the ability and trait emotional intelligence as predictors and grandiose narcissism (model 1), and vulnerable narcissism (model 2) as dependent variables.

	Model 1. Outcome: Grandiose narcissism				Model 2. Outcome: Vulnerable narcissism			
Predictor	Δ*R*^2^	β	*P*	*F*(2,248)	Δ*R*^2^	β	*P*	*F*(2,248)
Ability EI	0.03	−0.19	0.001	46.80; *p* < 0.001	0.01	−0.04	0.496	53.17; *p* < 0.001
Trait EI	0.26	0.52	0.000		0.29	−0.54	0.000	

*ΔR^2^, incremental R for each predictor when entered after the other predictor.*

## Discussion

We examined the relationship between narcissism and EI. The results suggest that to fully understand the complex relations between these constructs, the two types of narcissism as well as various conceptualizations of EI need to be taken into account. We have found that grandiose and vulnerable narcissisms were strongly associated with trait EI, although the correlations had different signs: positive and negative, respectively. Thus H1 and H2 were confirmed. Although we expected vulnerable narcissism to be negatively associated with ability EI (H2), both narcissisms did not significantly correlate with this type of EI. These results are consistent with some previous findings (Petrides et al., 2011; Vonk et al., 2013; Austin et al., 2014; Nagler et al., 2014; Zhang et al., 2015; Czarna et al., 2016; Jauk et al., 2016), however, the fact that we have included two different measures of EI allowed for further analyses, which provided us with especially interesting results discussed below.

Furthermore, we conducted regression models to test how the two narcissisms are uniquely associated with each EI measure controlling for the other EI. The zero-order correlations indicated that grandiose narcissism was positively correlated with trait EI, and weakly (non-significant) and negatively with ability EI, however, regression models revealed interesting suppression effects. A suppressor effect is observed when the validity coefficient of one variable is enhanced by the inclusion of another variable to the model (Paulhus et al., 2004). In our study, the associations of grandiose narcissism with the two EIs became more pronounced when the three variables were analyzed together. In all such models, grandiose narcissism was negatively and significantly related to ability EI. It seems then that when the common variance of the two EIs is removed, the discrepancy between grandiose narcissism’s relations with each EI becomes more visible. It is possible, that when one’s realistic perception of his/her EI is controlled, the positive illusions about EI might be observed. This result is in line with our H4 as well as other data showing that inflated self-views are one of the defining characteristics of grandiose narcissism (Campbell and Foster, 2007). Indeed, a number of studies have indicated that individuals scoring high on grandiose narcissism tend to overestimate their qualities, especially agentic features, such as cognitive ability. For instance, a positive correlation between narcissism and self-evaluated intelligence has been observed even after controlling for objectively measured cognitive ability (Gabriel et al., 1994; Paulhus and Williams, 2002; Zajenkowski and Czarna, 2015). Interestingly, it has been already shown that in the domain of emotion recognition, grandiose narcissists also overestimate their skills. In a recent study Lobbestael, et al. (2016) showed a lack of convergence between subjective and objective levels of emotional ability (i.e., the Reading the Mind in the Eye test vs. subjective opinion about emotional recognition skill). This finding as well as our results imply that narcissists think highly of themselves on a number of agentic domains including emotional ability. The question arises about the role of this inflated self-view. Our results appear to be in line with the extended agency model of narcissism according to which an inflated view of one’s own ability is an important strategy serving a self-regulatory function to maintain positive feelings (Campbell and Foster, 2007). Indeed, the self-enhancement of individuals with high grandiose narcissism was found to positively influence their well-being (Dufner et al., 2012). Moreover, prior work on intelligence indicate that self-enhancement may shape self-esteem in grandiose narcissism (Dufner et al., 2012). Thus, it would be interesting to include the latter variable in future studies to examine its role in the link between narcissism and the EI overestimation.

Vulnerable narcissism was negatively correlated with trait EI, however it was not related to global ability EI in any of the tested models. Moreover, the relationship between both EIs decreased when they were analyzed in one model with vulnerable narcissism. Thus individuals with high vulnerable narcissism perceive their emotional abilities as rather poor. People scoring high on vulnerable narcissism are concentrated on themselves, but at the same time, in contrast to grandiose narcissists, they exhibit depression and low self-esteem, and perhaps are less positively biased.

The present study also revealed that although vulnerable narcissism did not correlate with the global ability EI, it was relatively highly associated with the facilitation subscale from the ability EI measure. Facilitation reflects an ability to assimilate emotions with thinking and problem solving as well as to use emotion to direct attention to important information (Mayer et al., 2004). One may wonder to what extent the difficulties in this area might be responsible for the affective problems observed in vulnerable narcissism. Particularly, individuals high on vulnerable narcissism display a tendency toward negative emotionality including depression, anxiety, anger, little positive affect, and a substantial degree of negative affect (Miller et al., 2011). Additionally, they exhibit biased social perception in the form of increased levels of hostility and mistrust (Krizan and Johar, 2015). It is possible that low facilitation ability makes it difficult to disengage their thoughts from negative emotions, which in turn leads to biased information processing. However, this interpretation is rather speculative and further studies are necessary to examine the role of facilitation in vulnerable narcissism.

Some researchers suggest that narcissists (mainly grandiose) might exhibit an increased ability to read and assess others’ emotions, which is subsequently utilized to formulate strategies with which they can acquire what they want (Jonason and Kroll, 2015). Our study does not support this hypothesis about narcissism’s superior ability pertaining to emotion perception; however, it needs to be mentioned that neither type of narcissism showed severe deficits in this area, being only weakly associated with the perception subscale from the ability EI measure.

Finally, our study sheds some light on the nature of EI. We found that both types of narcissism were highly associated with trait EI and, trait EI and ability EI were only weakly correlated. This finding seems to be in accordance with the theoretical background and the empirical data on trait EI. Petrides (e.g., Petrides et al., 2007) emphasized that trait EI reflects people’s perceptions of their emotional abilities and thus is a part of personality assessed via self-report questionnaires. Indeed, trait EI is highly correlated with personality traits (e.g., Pearson’s *r*s exceeding 0.60 in the cases of extraversion and neuroticism), and much lower with ability EI (Petrides et al., 2007). Our results are consistent with this view and indicate that the two types of narcissism are important correlates of trait EI. These findings suggest that narcissism may play a substantial role in understanding EI at both the conceptual and measurement level.

Some evident limitations of our study should be noted. First, in the investigated sample females were overrepresented, while previous studies show that men have higher levels of grandiose narcissism (e.g., Grijalva et al., 2015) and women have higher EI (Mayer et al., 1999; Petrides and Furnham, 2000). Thus the difficulties in EI among narcissists might more pronounced in a more differentiated sample. Second, the present investigation was conducted via online survey and more controllable laboratory conditions, especially for performance EI, would increase the reliability of the results.

## Author Contributions

MZ and PU designed the study. PU conducted the study. MZ, OM, KS, and PU wrote the manuscript. MZ performed the analyses.

## Conflict of Interest Statement

The authors declare that the research was conducted in the absence of any commercial or financial relationships that could be construed as a potential conflict of interest. The reviewer EM and handling Editor declared their shared affiliation at the time of the review.
