# Corrigendum: Vulnerable and Grandiose Narcissism Are Differentially Associated With Ability and Trait Emotional Intelligence

**DOI:** 10.3389/fpsyg.2018.02392

**Published:** 2018-11-28

**Authors:** Marcin Zajenkowski, Oliwia Maciantowicz, Kinga Szymaniak, Paweł Urban

**Affiliations:** Faculty of Psychology, University of Warsaw, Warsaw, Poland

**Keywords:** grandiose narcissism, vulnerable narcissism, emotional intelligence, ability, narcissism

There is an error in the Funding statement. The correct number for National Science Centre in Poland is 2016/23/B/HS6/00312. The authors apologize for this error and state that this does not change the scientific conclusions of the article in any way. The original article has been updated.

## Conflict of interest statement

The authors declare that the research was conducted in the absence of any commercial or financial relationships that could be construed as a potential conflict of interest.

